# 

**Published:** 1806-08

**Authors:** 


### LIST OF NEW MEDICAL PUBLICATIONS.

Observations on the Nature, Kinds, Causes, and Prevention of Insanity. By Thomas Arnold, M. D. Fellow of the Royal College of Physicians, &c. the Second Edition, corrected and improved, in 2 Vols. Price 1l. 1s.

Dr. Willan, on Vaccine Inoculation, 4to.

Admonitory Hints on the Use of Sea Bathing. By J. Peake. 1s. 6d.

Observations, &c. on the late Epidemic Disease at Gibraltar; intended to illustrate the contagious Fevers in general. By S. H. Jackson, M. D. 5s. boards.

A Letter to Mr. Birch, in Answer to his late Pamphlet against Vaccination. 1s. 6d.

A Reply to the Anti-Vaccinists. By James Moore. 2s.

---

### *TO CORRESPONDENTS.*

Dr. Hamilton's Observations on Digitalis, with Cases of Hydrothorax, and Dr. Kinglake's paper, in Answer to Dr. Kentish, will appear in our next Number.

Communications are also received from Mr. Laurense, Mr. Atkinson, and Dr. Jardine.

